# Repurposing the Antibiotic Tigecycline to Inhibit Tumor Growth and Hormone Secretion in Somatotroph Pituitary Neuroendocrine Tumors

**DOI:** 10.1155/ije/3784550

**Published:** 2026-06-24

**Authors:** Zhiqian Yang, Yang Liu, Dapeng Wu, Bin Xu, Shengfa Zeng, Wei Zhang

**Affiliations:** ^1^ Department of Neurosurgery, The First Affiliated Hospital of Guangdong Pharmaceutical University, No. 19 Nonglinxia Road, Guangzhou, 510080, China, gdpu.edu.cn; ^2^ Guangdong Provincial Engineering and Technology Research Center of Stem Cell Therapy for Pituitary Disease, No. 19 Nonglinxia Road, Guangzhou, 510080, China; ^3^ Department of Neurosurgery, Huai’an Hospital, No. 19 Shanyang Road, Huai’an, 223220, China; ^4^ Center of Teaching Quality Monitoring and Assessment, Guangdong Pharmaceutical University, No. 280 Outer Ring East Road Guangzhou Higher Education Mega Center, Guangzhou, 510006, China, sysu.edu.cn

**Keywords:** Akt/mTOR signaling pathway, growth hormone, somatotroph pituitary neuroendocrine tumor (PitNET), tigecycline

## Abstract

**Objective:**

This investigation employed the rat GH3 somatotroph pituitary neuroendocrine tumor (PitNET) cell line to assess the effects of the antibiotic tigecycline and to preliminarily elucidate its potential molecular mechanisms.

**Methods:**

GH3 cells were exposed to tigecycline ranging from 6.25 to 100 μM. Cell viability and IC_50_ were determined using the CCK‐8 assay, and spheroid growth was monitored by measuring diameters. To assess apoptosis, cells were subjected to Annexin V‐FITC/PI staining and nuclear morphology observation after DAPI staining. Meanwhile, the cell cycle distribution was profiled via flow cytometry following propidium iodide (PI) staining. The expression of proteins related to the Akt/mTOR pathway, apoptosis, and growth hormone (GH) was evaluated by Western blot. GH secretion was quantified using ELISA.

**Results:**

Tigecycline dose and time dependently suppressed GH3 cell proliferation, with IC_50_ values of 22.45 μM at 48 h and 9.037 μM at 72 h. It also impeded three‐dimensional spheroid growth. Mechanistically, treatment induced G0/G1 phase arrest, significantly suppressed the Akt/mTOR signaling pathway (evidenced by reduced phosphorylation of Akt and mTOR), and activated the intrinsic apoptotic pathway, marked by a rise in the Bax/Bcl‐2 ratio and heightened cleaved caspase‐3 expression. Additionally, tigecycline significantly attenuated both intracellular synthesis and extracellular secretion of GH.

**Conclusion:**

These findings indicate that tigecycline exerts potent antiproliferative and antisecretory effects on GH3 cells, likely through Akt/mTOR pathway inhibition, cell cycle arrest, and apoptosis induction. These results provide an experimental foundation for considering tigecycline as a potential therapeutic agent for GH‐secreting PitNET.

## 1. Introduction

Pituitary neuroendocrine tumors (PitNETs), formerly known as pituitary adenomas, represent the most prevalent neoplasms of the sellar region. Beyond inducing secondary hypopituitarism through mass effects that compress pituitary tissue and surrounding vasculature, these tumors can lead to systemic metabolic disturbances and end‐organ damage due to excessive hormone secretion. Pharmacotherapy serves as a crucial treatment modality, often employed alongside surgical intervention [1]. Clinically available drugs primarily fall into three categories: somatostatin analogs (e.g., octreotide), agonists of dopamine receptors (e.g., bromocriptine and cabergoline), and antagonists of the growth hormone (GH) receptor (e.g., pegvisomant) [2]. While these agents are effective in controlling hormone levels and shrinking tumor mass in many patients, a subset exhibits significant drug resistance or experience adverse effects, resulting in suboptimal therapeutic outcomes [3].

Tigecycline, a broad‐spectrum antibiotic widely used in clinical practice [4], has recently attracted attention for its potential antitumor properties. Evidence of its efficacy has been reported in several tumor models, such as pancreatic ductal adenocarcinoma [5], sorafenib resistance in hepatocellular carcinoma [6], and multiple myeloma [7].

However, its role in intracranial tumors, particularly PitNETs, remains largely unexplored. This research aimed to comprehensively evaluate the effects of tigecycline on GH3 cells, encompassing proliferation, cell cycle, apoptosis, and hormone secretion. A parallel objective was to decipher the molecular mechanisms underlying these effects, focusing on the Akt/mTOR pathway and apoptotic regulators. The findings may offer novel perspectives for drug repurposing and therapeutic strategy development in somatotroph PitNETs.

## 2. Materials and Methods

### 2.1. Cell Cultivation System and Treatment Protocols

GH3 cells, purchased from National BioResource Cell Center, were maintained in Kaighn’s modified Ham’s F‐12 nutrient mixture containing 10% FBS in a humidified 5% CO_2_ atmosphere. Logarithmically growing cells were then exposed to a gradient of tigecycline concentrations (6.25–100 μM), while control cells received vehicle only.

### 2.2. Cell Viability Assessment

The CCK‐8 assay was conducted to study the cell viability. After the indicated treatment periods, cell viability was determined by adding CCK‐8 reagent and incubating for 2 h, and then the absorbance at 450 nm was recorded using a microplate reader. Additionally, after 48 and 72 h of tigecycline treatment, three random areas per well were selected, and the diameters of spheroids larger than 30 μm formed by GH3 cells were measured using ImageJ software. Spheroids with a diameter ≥ 30 μm were selected for analysis as they represent structurally stable and proliferatively active multicellular aggregates, minimizing counting bias from cellular debris or small clumps.

### 2.3. Cell Cycle Analysis

GH3 cells were harvested after 48 or 72 h of tigecycline treatment. Their cell cycle profiles were then analyzed through flow cytometric analysis subsequent to staining with propidium iodide (PI).

### 2.4. Apoptosis Assay

Flow cytometry: To assess apoptosis, cells were stained using an Annexin V‐FITC and PI double staining kit (C1062, Beyotime Biotechnology, Shanghai) and subjected to flow cytometric analysis. Before this procedure, GH3 cells had been treated with either 6.25 or 25 μM tigecycline for 48 or 72 h.

DAPI staining: For the morphological evaluation of apoptosis, the cells were subjected to sequential PBS washing, 4% paraformaldehyde fixation, and DAPI staining. Characteristic nuclear changes indicative of apoptosis were identified using fluorescence microscopy, and the apoptosis rate was calculated accordingly.

### 2.5. Western Blot Analysis

Following extraction, protein concentration of cell lysates was quantified using the BCA assay. Total proteins were resolved via SDS‐PAGE and electroblotted onto PVDF membranes. Following a blocking step using 5% BSA, the membranes were incubated overnight at 4°C with the respective primary antibodies. Antibodies from Proteintech targeted phospho‐Akt (Ser473; 66444‐1‐Ig), total Akt (60203‐2‐Ig), and Bcl‐2 (26593‐1‐AP). Antibodies from Cell Signaling Technology (CST) recognized phospho‐mTOR (Ser2448; 5536), total mTOR (2983), and cleaved caspase‐3 (9661). The remaining primary antibodies used were directed against Bax (ab32503, Abcam), GH (ab230996, Abcam), and GAPDH, which served as the loading control. Visualization and quantification of protein bands were achieved by enhanced chemiluminescence (ECL) detection (FD8020, FUDE Biotechnology) and subsequent ImageJ densitometry, respectively, following incubation with HRP‐conjugated secondary antibodies.

### 2.6. ELISA

GH secretion from GH3 cells was assessed by measuring its concentration in the culture supernatant with a rat‐specific sandwich ELISA kit (Cusabio, CSB‐E07342r). The manufacturer’s guidelines were adhered to during all experimental procedures.

### 2.7. Statistical Analysis

To evaluate statistical significance, data were assessed using one‐way ANOVA/Dunnett’s post hoc test for multiple group comparisons in GraphPad Prism. Data are shown in the form of mean ± SD. Results were deemed statistically significant when the *p* value fell below 0.05. In all figures, statistical significance is indicated as follows: ^∗^ *p* < 0.05, ^∗∗^ *p* < 0.01, ^∗∗∗^ *p* < 0.001, and ^∗∗∗∗^ *p* < 0.0001.

## 3. Results

### 3.1. Tigecycline Suppresses GH3 Cell Proliferation and Spheroid Growth

**FIGURE 1:**
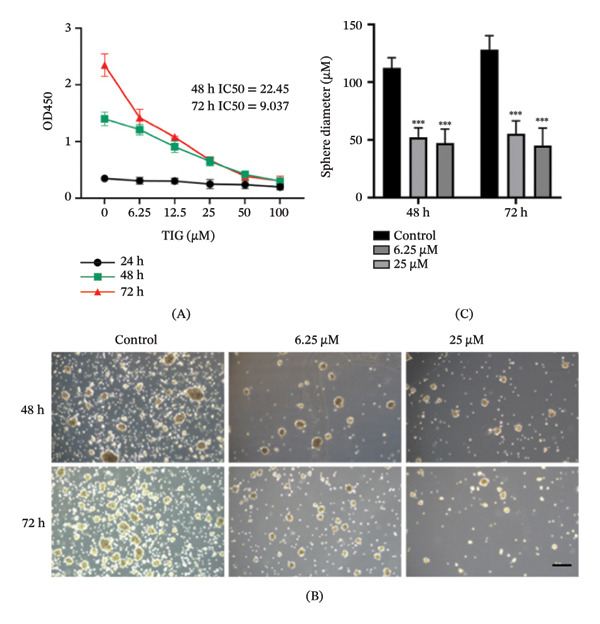
Tigecycline (TIG) suppresses proliferation and growth of GH3 cells. (A) OD450 of GH3 from different groups was measured by the CCK‐8 assay after being exposed to specified TIG concentrations for 24, 48, and 72 h. The calculated half‐maximal inhibitory concentrations (IC_50_) for the 48‐h and 72‐h time points are indicated. (B) Representative microscopic images of GH3 cell spheroids treated with TIG for 48 and 72 h. (C) Quantification of the diameters of spheroids larger than 30 μm from (B). Values represent the mean ± SD (*n* = 5). Differences relative to the control were evaluated by one‐way ANOVA and Dunnett’s post hoc test. ^∗∗∗^ *p* < 0.001.

As shown in Figure 1A, the CCK‐8 assay demonstrated that tigecycline significantly suppressed the viability of GH3 cells in a concentration‐ and time‐dependent manner over 24–72 h, with IC_50_ values of 22.45 μM (48 h) and 9.037 μM (72 h).

Consistent with this, microscopic examination showed a substantial reduction in cell spheroid size upon tigecycline exposure compared to the untreated control (Figure 1B). Quantitative analysis confirmed a significant decrease in the average diameter of spheroids larger than 30 μm. The mean diameters were as follows: 112.24 μm (control) vs. 52.35 μm (6.25 μM) vs. 47.23 μm (25 μM) at 48 h; and 128.25 μm (control) vs. 55.52 μm (6.25 μM) vs. 45.12 μm (25 μM) at 72 h (Figure 1C).

### 3.2. Tigecycline Triggered G0/G1 Phase Arrest in GH3

**FIGURE 2:**
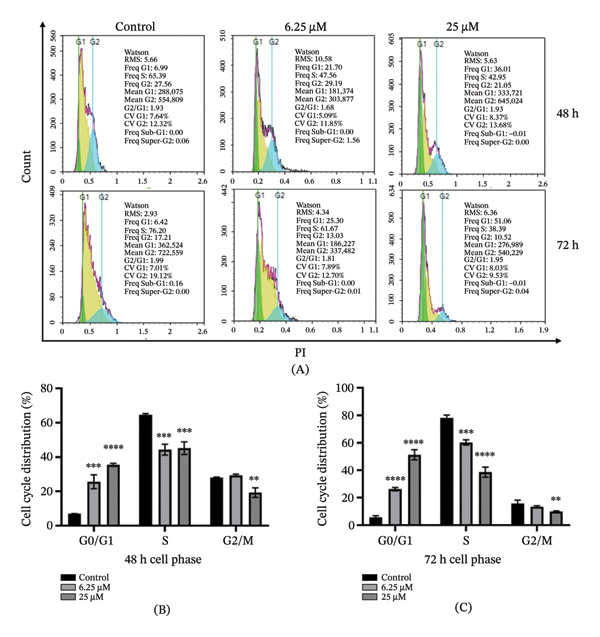
Tigecycline triggers G0/G1 arrest in GH3 cells. (A) Representative flow cytometry plots of PI‐stained GH3 cells following treatment with 6.25 or 25 μM TIG for 48 and 72 h. (B) Profile of cell cycle distribution at the 48‐h time point. (C) Cell cycle distribution after 72 h TIG treatment. Values represent the mean ± SD (*n* = 3). Differences relative to the control group were evaluated by one‐way ANOVA and Dunnett’s post hoc test. Significance markers are defined as follows: ^∗∗^ *p* < 0.01, ^∗∗∗^ *p* < 0.001, and ^∗∗∗∗^ *p* < 0.0001.

GH3 cells were exposed to tigecycline for 48 or 72 h to assess its impact on the cell cycle. As shown in Figure 2, tigecycline treatment dose dependently elevated the proportion of GH3 in the G0/G1 phase, while simultaneously decreasing the percentages in the S and G2/M phases relative to controls. This G0/G1 phase accumulation suggests that tigecycline inhibits GH3 cell proliferation through cell cycle arrest.

### 3.3. Tigecycline‐Mediated Inhibition of the Akt/mTOR Cascade

**FIGURE 3:**
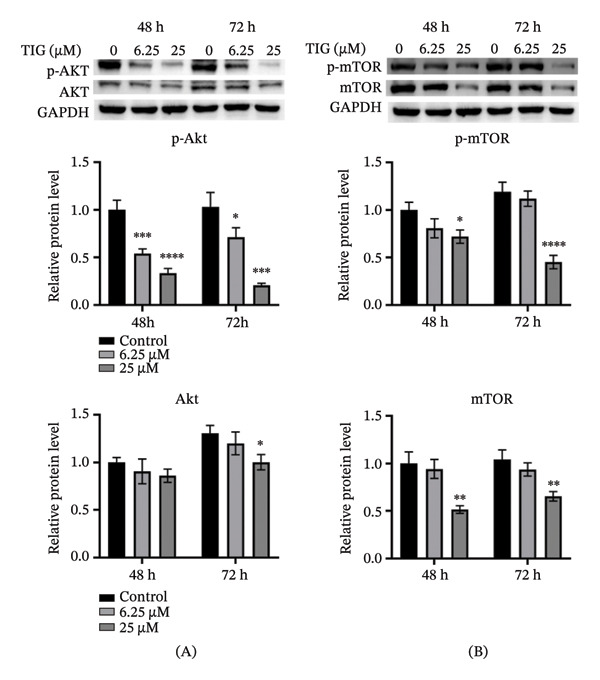
Tigecycline suppresses the Akt/mTOR cascade in GH3. (A, B) Western blot detection of phosphorylated and total Akt and mTOR in GH3 cells exposed to 6.25 or 25 μM TIG for 48 and 72 h. Protein levels were quantified by densitometric analysis (ImageJ) and are reported in the form of mean ± SD (*n* = 3). Differences relative to the control group were evaluated by one‐way ANOVA and Dunnett’s post hoc test. Significance markers are defined as follows: ^∗^ *p* < 0.05, ^∗∗^ *p* < 0.01, ^∗∗∗^ *p* < 0.001, and ^∗∗∗∗^ *p* < 0.0001.

In order to unravel the mechanistic basis, the impact of tigecycline on the Akt/mTOR pathway was examined via Western blot. While 6.25 μM tigecycline over 48 or 72 h had minimal effect on total Akt/mTOR protein levels, treatment with 25 μM markedly reduced both total and phosphorylated forms. Importantly, phosphorylation of both Akt and mTOR (p‐Akt and p‐mTOR) was significantly suppressed by tigecycline at both concentrations, with this inhibitory effect being more evident upon extended exposure (Figure 3). Collectively, these findings demonstrate that tigecycline effectively inhibits Akt/mTOR signaling in GH3 cells.

### 3.4. Tigecycline Promotes Apoptosis in GH3 Cells

**FIGURE 4:**
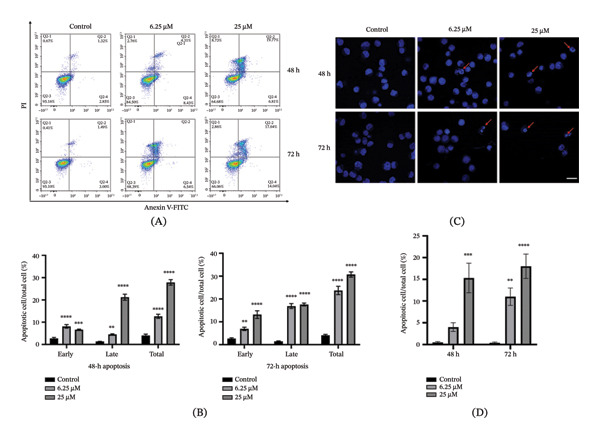
Tigecycline triggers apoptosis in GH3 cells. (A) Flow cytometry dot plots shows Annexin V‐FITC/PI‐stained GH3 cells after exposure to 6.25 or 25 μM TIG for 48 and 72 h. (B) Quantitative analysis of the total apoptotic cell percentage from (A). (C) Fluorescence microscopy images of DAPI‐stained nuclei; arrows highlight apoptotic morphology. Scale bar = 20 μm. (D) The percentage of GH3 with pyknotic nuclei from (C). Data are given in the form of mean ± SD (*n* = 3). Differences relative to the control group were evaluated by one‐way ANOVA and Dunnett’s post hoc test. Significance markers are defined as follows: ^∗∗^ *p* < 0.01, ^∗∗∗^ *p* < 0.001, and ^∗∗∗∗^ *p* < 0.0001.

The induction of apoptosis by tigecycline in GH3 was assessed by Annexin V‐FITC/PI double staining. The results demonstrated that tigecycline elicited apoptotic cell death in a manner dependent on both concentration and duration of exposure. Based on Annexin V/PI staining profiles, flow cytometric profiles showed a progressive decline in the viable cell population with increasing drug concentration, alongside corresponding increases in early apoptotic, late apoptotic, and necrotic cell fractions (Figure 4A,B). For example, treatment with 6.25 μM tigecycline resulted in an increase in Annexin V–positive cells from 12.6% ± 0.92% at 48 h to 23.73% ± 1.82% at 72 h (Figure 4B).

Morphological assessment via DAPI staining further confirmed apoptosis induction. Tigecycline‐treated cells exhibited characteristic apoptotic features, such as nuclear condensation, fragmentation, and pyknosis, which were minimal in control cells (Figure 4C). Quantitative analysis showed that treatment with 6.25 and 25 μM tigecycline for 72 h significantly increased the proportion of cells with pyknotic nuclei (Figure 4D).

### 3.5. Tigecycline Activates the Caspase‐3 Apoptotic Pathway in GH3 Cells

**FIGURE 5:**
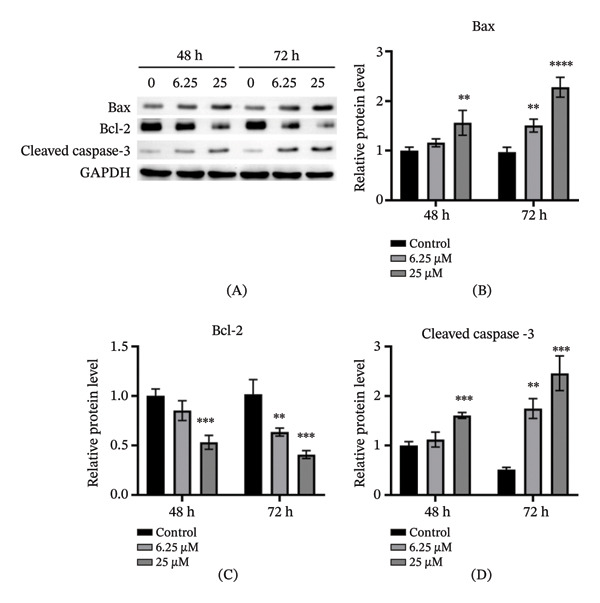
Tigecycline activates Bax/Bcl‐2/caspase‐3 axis in GH3. (A) The expression of Bcl‐2, Bax, and cleaved caspase‐3 in GH3 cells exposed to TIG (6.25 or 25 μM) for 48 or 72 h was evaluated by Western blot. (B–D) Quantitative analysis of Bcl‐2, Bax, and cleaved caspase‐3 expression. Data are presented as mean ± SD (*n* = 3). Differences relative to the control were evaluated by one‐way ANOVA and Dunnett’s post hoc test. Significance markers are defined as follows: ^∗∗^ *p* < 0.01, ^∗∗∗^ *p* < 0.001, and ^∗∗∗∗^ *p* < 0.0001.

To profile apoptotic signaling, we examined key regulatory proteins by Western blot. Tigecycline treatment concentration and time dependently increased the expression of the proapoptotic effector Bax and activated caspase‐3 (cleaved form), while decreasing the antiapoptotic protein Bcl‐2 (Figure 5). This expression pattern correlates with activation of the intrinsic mitochondrial apoptotic pathway, implicating the Bcl‐2/Bax balance and downstream caspase‐3 cleavage in tigecycline‐induced apoptosis of GH3 cells.

### 3.6. Tigecycline Inhibits GH Synthesis and Secretion

**FIGURE 6:**
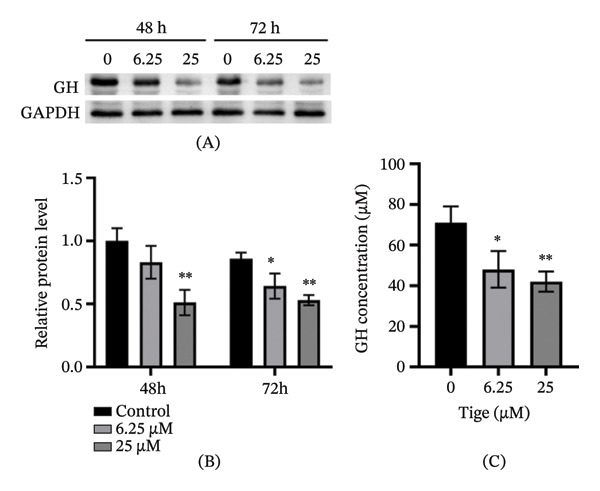
Tigecycline reduces GH synthesis and secretion in GH3 cells. (A) Representative immunoblots of intracellular GH after treatment with indicated TIG concentrations for 48 and 72 h. (B) Densitometric analysis of GH protein levels from (A). (C) Measurement of secreted GH in the supernatant by ELISA after 72 h treatment. Data are presented as the mean ± SD (*n* = 3). Differences relative to the control were evaluated by one‐way ANOVA and Dunnett’s post hoc test. Significance markers are defined as follows: ^∗^ *p* < 0.05 and ^∗∗^ *p* < 0.01.

Given the secretory function of GH3, we studied the effect of tigecycline on GH production. Western blot analysis revealed that tigecycline treatment for 48 or 72 h significantly reduced intracellular GH protein levels in a concentration‐dependent manner (Figure 6A,B). Consistent with this, ELISA measurements confirmed a decrease in GH concentration in the culture supernatant of TIG‐treated GH3 (Figure 6C). Together, these data support that tigecycline suppresses both the synthesis and secretion of GH in GH3 cells.

## 4. Discussion

Our in vitro data establish the multifaceted antitumor properties of tigecycline against the rat GH3 somatotroph PitNET cell line. The compound potently curbs cell proliferation, arrests the cell cycle, induces programmed cell death, and attenuates GH synthesis. These biological effects are primarily mediated through the Akt/mTOR signaling cascade inhibition and the subsequent activation of the mitochondrial apoptotic cascade.

A dose‐ and time‐dependent suppression of GH3 cell viability was observed following tigecycline treatment. This antiproliferative effect was further supported by a significant reduction in spheroid size, underscoring its growth‐suppressive activity. In this study, spheroids with a diameter ≥ 30 μm were selected for analysis to ensure the inclusion of structurally stable and proliferatively active multicellular aggregates, thereby minimizing counting bias from cellular debris or small, nonrepresentative clumps. Given that GH3 cells exhibit semiadherent and semisuspension growth characteristics, this threshold‐based approach provides a practical and reproducible means of quantifying tigecycline’s antiproliferative effects. However, this methodological choice may introduce selection bias by excluding smaller spheroids that could also reflect treatment‐induced growth inhibition. Moreover, the threshold of 30 μm, while commonly used in spheroid‐based assays, lacks a standardized definition and may vary across studies, potentially limiting cross‐study comparability. Future investigations should consider employing automated imaging platforms coupled with unbiased image analysis algorithms (e.g., CellProfiler) to capture and analyze spheroids across the full size spectrum, thereby enabling a more comprehensive assessment of antitumor efficacy. Our results align with prior studies demonstrating the anticancer potential of tigecycline in diverse malignancies such as acute myeloid leukemia [8], pancreatic ductal adenocarcinoma [5], and liver cancer [9]. The consistent dose–response relationship across models reinforces the therapeutic promise of tigecycline.

At the molecular level, the Akt/mTOR pathway was identified as a principal target of tigecycline. Treatment with 25 μM tigecycline selectively reduced phosphorylation of Akt (Ser473) and mTOR (Ser2448) without affecting total protein levels, suggesting a specific inhibition of pathway activation rather than general protein expression. Since constitutive Akt/mTOR signaling is a known driver in PitNETs pathogenesis, especially in GH‐secreting subtypes [10, 11], these suggest that the antitumor activity of tigecycline in GH3 cells is predominantly mediated through the suppression of this critical pathway.

Beyond suppressing proliferation, tigecycline robustly induced apoptosis in GH3 cells. Flow cytometry confirmed a marked elevation in the apoptotic cell fraction, a finding corroborated by DAPI staining revealing characteristic nuclear condensation and fragmentation. At the molecular level, tigecycline triggered a proapoptotic rebalancing of the Bcl‐2 family, evidenced by an elevated Bax/Bcl‐2 ratio due to concurrent Bax upregulation and Bcl‐2 downregulation. This shift was accompanied by the cleavage of caspase‐3, confirming the activation of the mitochondrial apoptotic pathway. The alignment of this mechanism with reports in tigecycline‐treated leukemia stem cells [12] indicates that tigecycline activates a conserved apoptotic program across diverse cancer types.

Tigecycline also induced a marked G0/G1 phase arrest in GH3 cells, a response that diverges from its effects in some melanoma lines [13], underscoring cell‐type specificity. Given the established role of the Akt/mTOR signaling axis in governing the G1/S transition via regulators such as cyclin D1 and p21 [14], we propose that the observed arrest may therefore stem from tigecycline’s inhibition of this signaling axis. Direct assessment of these downstream cell cycle proteins in future work would help substantiate this model.

A therapeutically relevant finding of this study is the observed suppression of GH synthesis and secretion by tigecycline. In GH‐secreting PitNETs, achieving biochemical control is as critical as inhibiting tumor growth. Current first‐line medical therapies, such as somatostatin analogs, primarily target hormone secretion. The ability of tigecycline to concurrently suppress both proliferation and hormone production suggests a potential dual mechanism of action, which could offer a distinct therapeutic advantage for patients with acromegaly.

To date, no clinical cases have been reported regarding the effect of tigecycline on GH or IGF‐1 levels in patients with PitNETs. However, our in vitro findings suggest a potential endocrine effect that warrants investigation in future clinical studies or retrospective analyses.

Regarding the clinical significance of repurposing tigecycline, as an FDA‐approved antibiotic with well‐characterized pharmacokinetics and safety profiles, tigecycline offers a favorable starting point for drug repurposing. Notably, the observed antiproliferative and antisecretory effects in GH3 cells were achieved at concentrations (48‐h IC50 = 22.45 μM, equivalent to 13.15 μg/mL) that are higher than standard clinical serum concentrations (0.7–1.9 μg/mL) following conventional dosing regimens [15, 16]. However, higher dosing regimens (e.g., 200–400 mg loading dose followed by 100–200 mg once daily) can achieve peak serum concentrations [17], and tigecycline is known to accumulate in certain tissues at levels exceeding those in serum [15]. These findings suggest that dose optimization or localized delivery strategies may be required to achieve antitumor efficacy in clinical settings. A legitimate concern is whether the observed effects reflect general cytotoxicity or specific antitumor activity. The induction of G0/G1 arrest, modulation of the Akt/mTOR pathway, and suppression of GH synthesis argue against nonspecific toxicity. Furthermore, the antitumor properties of tigecycline are attributed to its unique ability to inhibit mitochondrial translation [15], a mechanism not shared by other antibiotics such as vancomycin. Thus, the effects observed are likely specific to tigecycline. Future in vivo studies and comparative assessments with other antibiotics are warranted to confirm these findings.

Certain limitations must be considered when interpreting the findings of this study. First, all conclusions are derived from in vitro experiments; thus, the efficacy and safety of tigecycline must be validated in in vivo models. Second, the upstream mechanisms by which tigecycline inhibits Akt/mTOR signaling remain unclear. Whether this involves disruption of mitochondrial function, induction of endoplasmic reticulum stress, or interference with amino acid metabolism warrants further investigation. Finally, as an antibiotic, the potential systemic toxicity and the blood–brain barrier permeability of tigecycline are critical considerations for its repurposing as an anticancer agent for pituitary tumors.

The present study establishes that tigecycline possesses significant antitumor and antisecretory activity against GH3 somatotroph PitNET cells. Its mechanism primarily involves suppression of the Akt/mTOR pathway, leading to cell cycle arrest, apoptotic induction, and reduced GH production. Taken together, the dual inhibition of tumor growth and hormone secretion provides compelling evidence for advancing tigecycline as a promising candidate in the therapeutic strategy for GH‐secreting PitNETs.

## 5. Conclusion

Collectively, our data show that tigecycline exhibits robust antitumor activity against GH3 somatotroph PitNET cell line, primarily by curtailing proliferation, inducing G0/G1 arrest, and initiating the Bcl‐2/Bax/caspase‐3‐mediated mitochondrial apoptotic cascade. Mechanistically, these effects are linked to inhibition of the Akt/mTOR signaling axis and a concomitant decrease in GH production. This work thus provides a preclinical foundation for repurposing tigecycline as a potential therapeutic agent for GH‐secreting PitNETs. Subsequent studies should prioritize in vivo validation and the elucidation of upstream regulatory mechanisms to facilitate its translational progression.

## Funding

This study was funded by the Guangdong Provincial Health Commission, B2025417; National Key Research and Development Program of China, 2024YFC3406204.

## Conflicts of Interest

The authors declare no conflicts of interest.

## Data Availability

The data that support the findings of this study are available from the corresponding author upon reasonable request.
